# Two cases of AMeD syndrome with isochromosome 1q treated with allogeneic stem cell transplantation

**DOI:** 10.1016/j.lrr.2024.100476

**Published:** 2024-07-24

**Authors:** Mari Kagajo, Kyoko Moritani, Mayumi Iwamoto, Machiko Miyamoto, Tsuyoshi Imai, Motoharu Hamada, Manabu Wakamatsu, Hideki Muramatsu, Minenori Eguchi-Ishimae, Mariko Eguchi

**Affiliations:** a Department of Pediatrics, Ehime University Graduate School of Medicine, Japan; b Department of Pediatric Hematology and Oncology, NHO Shikoku Medical Center for Children and Adults, Japan; c Department of Virology, Nagoya City University Graduate School of Medical Sciences, Japan; d Department of Pediatrics, Nagoya University Graduate School of Medicine, Japan

**Keywords:** AMeD syndrome, Inherited bone marrow failure, Hematopoietic stem cell transplantation, Isochromosome 1q

## Abstract

AMeD syndrome is characterized by aplastic anemia, mental retardation, short stature, and microcephaly and is caused by digenic mutations in the aldehyde dehydrogenase 2 (ALDH2) and alcohol dehydrogenase 5 (ADH5) genes. We have successfully performed hematopoietic stem cell transplantation in two patients with AMeD syndrome and isochromosome 1q. AMeD syndrome with myelodysplastic syndrome or acute myeloblastic leukemia generally has a poor prognosis; however, early diagnosis may improve treatment response. Although the gain of 1q has been considered as a form of early clonal evolution in Fanconi anemia, it may be an equally important finding observed in AMeD syndrome.

## Highlights

• It is known that AMeD syndrome with MDS or AML usually has a poor prognosis.
• We report two cases of AmeD syndrome with isochromosome 1q successfully treated with hematopoietic cell transplantation.
• This gain of 1q may be considered a form of early clonal evolution, similar to Fanconi anemia, and could be useful in diagnosing AMeD syndrome.

## 1 Introduction

Inherited bone marrow failure syndrome (IBMFS) is a family of rare genetic disorders that is characterized by bone marrow (BM) failure with unique phenotypes and predisposition to cancer. Comprehensive genetic analyses have been conducted to diagnose classic IBMFSs, and advances in molecular genetics have led to the identification of disorders, such as AMeD syndrome or aldehyde degradation deficiency (ADD) syndrome [1,2]. AMeD syndrome is characterized by aplastic anemia, mental retardation, short stature, and microcephaly. This syndrome is caused by digenic mutations in *ALDH2* and *ADH5*. Cytological and animal studies have shown that simultaneous loss of *ALDH2* and *ADH5* functions is associated with an increase in cellular formaldehyde sensitivity and multisystem abnormalities, including hematopoietic failure. It has been suggested that endogenous formaldehyde can drive the phenotypic expression of Fanconi anemia (FA) [3,4]. Hematopoietic stem cell transplantation (HSCT) is commonly performed for the treatment of AMeD syndrome; however, the timing and preconditioning regimens for HSCT have not been established.

In this study, we report two cases of AmeD syndrome with isochromosome 1q successfully treated with HSCT.

### 1.1 Case 1

The first case was a 15-month-old girl. She was born at 38 weeks of gestational age with a birth weight of 2560 g (–1.85 SD score). She underwent medical examination because of poor weight gain and possible developmental abnormalities. At the age of 9 months, she was admitted to the hospital for coronavirus disease 2019 (COVID-19) with seizures and pancytopenia, subsequently becoming transfusion-dependent. She was suspected to have IBMFS with trilineage hypocellular marrow accompanied by developmental delay, short stature (62 cm, −3.4 SD score), microcephaly (38.5 cm, −3.6 SD score), and café-au-lait spots. Genetic testing by whole exome sequencing indicated homozygosity for *ADH5* (NM_000671.4: c. 966delG, p.W322X) and *ALDH2* (NM_000690.4:c.G1510A, p.E504K). Following a transient elevation of the trilineage blood cell, BM examination revealed normal cellular marrow; however, cytogenetic analysis, which was performed twice after 14 months of age, revealed the presence of isochromosome 1q in 1 of the 20 metaphases in both examination (Table 1, P15). HSCT from an HLA-matched sibling donor was successful at the age of 1 year and 4 months. Fludarabine (Flu, 125 mg/m^2^), melphalan (L-PAM, 140 mg/m^2^), and total body irradiation (TBI, 3 Gy) were administered as a preconditioning regimen for HSCT. BM examination was performed on day 30 after HSCT, and the results of the karyotyping revealed 46, XX in 20 metaphases. Moreover, STR examination indicated a complete donor type with no graft-versus-host disease. One year after treatment, chromosome 1 abnormality was not observed.

**Table 1:** Clinical and cytogenetic data of AMeD syndrome.

	ADH5mutations	ALDH2 mutations	MDS/AML	Karyotype	HSCT	Prognosis (age, or months after HSCT)	Reference
P1	p.W322X p.W322X	p.E504K p.E504K	–	45,XX,−7 [8/10]	No	Died from interstitial pneumonia (9 years-old)	[1]
P2	p.W322X p.W322X	p.E504K p.E504K	AML	46, XX. der(5;17) (p10;q10),+8 [4/20]	Yes	Died after HSCT (3 years-old)	[1]
P3	p.W322X p.A278P	p.E504K WT	RCMD (MDS-MLD)	45,XX,+1,der(1;21)(q10;q10),−7[12/20] 46,idem,+7,add(18)(p11.2) [8/20]	Yes	Alive after second HSCT (14 years-old)	[1]
P4	p.W322X p.W322X	p.E504K WT	RAEB-1 (MDS-EB-1)	46,XX,ins(1;?)(q12;?)[2/20] 45,idem,add(7)(p11.2),−8, add(19)(p13) [14/20]	Yes	Alive after HSCT	[1]
P5	p.W322X p.W322X	p.E504K p.E504K	RCMD (MDS-MLD)	46,XY,add(7)(p15) [1/20]	No	Died from an infection (2 years-old)	[1]
P6	p.W322X p.A278P	p.E504K WT	RAEB-1 (MDS-EB-1)	46,XX,+1,der(1;22)(q10;q10) [2/20] 47, idem,del(7)(q?),add(17)(p11.2),+mar1 [9/20]	Yes	Alive after HSCT (12 years-old)	[1]
P7	p.W322X p.A278P	p.E504K WT	RAEB-1 (MDS-EB-1)	46,XY,+1,der(1;15)(q10;q10),add(17)(p11.2) 46,idem,add(17)	ND	Alive (16 years-old)	[1]
P8	p.L188PfsX4 p.A278P	p.E504K WT	–	–	ND	ND	[2]
P9	p.W322X p.A278P	p.E504K WT	–	–	ND	ND	[2]
P10	p.W322X p.W322X	p.E504K WT	–	–	ND	ND	[2]
P11	p.W322X p.G311D	p.E504K WT	AA progressing to RCMD (MDS)	46,XX,der(22)t(1;22)(q12;q13), der(22)t(1;22)(q12;q13) [20]	Yes	Alive after HSCT (59 months post-HSCT)	[2]
P12	p.W322X p.A278P	p.E504K WT	AA progressing to AML	46,XY,+1,der(1;15)(q10:q10), del(7)(q?),add(11)(q23) [19/20] 46,XY [1/20]	Yes	Died after HSCT (60 months post HSCT)	[2]
P13	p.W322X p.W322X	p.E504K WT	AA with RCMD (MDS)	46,XX,der(14)t(1;14)(q12;p11.2),der(21)t(1;21)(q12;p11.2) [19/20]	Yes	Alive after second HSCT (6 months post HSCT)	[2]
P14	p.W322X p.A278P	p.E504K WT	AA with RAEB2 (MDS)	46,XX,+1,der(1;7)(q10;p10) [20/20]	Yes	Alive	[2]
P15	p.W322X p.W322X	p.E504K p.E504K	Bone marrow failure	47, XX, +*i*(1)(q10) [1/20] 46,XX [19/20]	Yes	Alive after HSCT (1years post-HSCT)	case 1
P16	p.W322X p.A278P	p.E504K WT	RAEB-1 (MDS-EB-1)	48, XX, +*i*(1)(q10), add(9)(p22),add(14)(q32)x2,+19 [15/20] 48,idem,t(5;22)(p15;q11.2),add(8)(p11.2),−19.+mar1 [2/20] 48,idem,del(6)(q?),del(11)(q?),add(12)(p13),+14,-add(14),add(17)(p11.2),−18,add(21)(p11.2),+mar2 [3/20]	Yes	Alive after second HSCT (4 years post-HSCT)	case 2

MDS: myelodysplastic syndrome, AML: acute myeloblastic leukemia, HSCT: hematopoietic stem cell transplantation.

ND: No data, RCMD: refractory cytopenia with multilineage dysplasia, RAEB-1: refractory anemia with excess blasts-1.

RAEB-2: refractory anemia with excess blasts-2, MLD: multilineage dysplasia, RCC: refractory cytopenia of childhood, AA: anaplastic anemia.

### 1.2 Case 2

The second case was a 17-year-old girl. She was born at 41 weeks of gestational age with a birth weight of 3098 g (–0.09 SD). Her height at 5 years was 97.2 cm (–2.98 SD). Hence, she was followed-up for short stature. At 7 years old, she presented with thrombocytopenia, and was diagnosed with myelodysplastic syndrome (MDS) with excess blasts accompanied by trilineage dysplasia and elevated blasts. The karyotyping revealed chromosomal abnormalities associated with the isochromosome1q (Table 1, P16). HSCT from an HLA-matched sibling donor was performed at 12 years of age. Flu (150 mg/m^2^), cyclophosphamide (CY, 40 mg/kg), anti-thymocyte globulin (ATG, 5 m/kg), and TBI (4 Gy) were administered as a preconditioning regimen according to the FA regimen for HSCT. Four months after HSCT, the patient developed mixed chimerism and was diagnosed with secondary graft failure. Then, HSCT from an unrelated 7/8 HLA allele-matched donor was performed at the age of 13 years. As a conditioning, Busulfan (BU, 16 mg/kg), CY (120 mg/kg), and L-PAM (140 mg/m^2^) were administered as per the MDS regimen [5]. Although engraftment of neutrophils was successful, her thrombocyte levels did not return to normal. Thrombopoietin receptor agonist (TPO-RA) was administered to improve persistent thrombocytopenia and was successfully discontinued after 1 year, as an increase in platelet count was observed. Because she presented with chromosomal abnormalities, developmental delay, short stature, microcephaly, and MDS, whole exome sequencing was performed using nail samples at the age of 17. The test revealed compound heterozygosity for *ADH5* (p.W322X and c.G832C, p.A278P) and heterozygosity of *ALDH2* (p.E504K). The patient showed good progress four years after the second HSCT.

## 2 Discussion

AmeD syndrome may progress from BM failure to MDS and acute myeloblastic leukemia (AML) [1]. The prognosis is poor once AML is developed [1]. Moreover, a good preconditioning regimen for HSCT to treat this disease has not established. In Case 2, the patient underwent HSCT with a preconditioning regimen for FA. Unfortunately, the patient experienced graft failure. In our cases myeloablative conditioning (MAC) regimens, such as BU/CY and FLU/MEL/TBI, have resulted in engraftment, suggesting its efficacy in treating AMeD syndrome. Therefore, a definitive diagnosis of AMeD syndrome prior to transplantation is helpful before considering a pretransplant regimen.

AMeD syndrome is caused by digenic mutations in biallelic *ADH5* and at least one allele of *ALDH2*. When ALDH2 is mutated in both allele (biallelic mutation), short stature and developmental delay tend to be more severe than cases with heterozygous, monoallelic mutations of *ALDH2* [1]. Three such cases have been reported, wherein all developed MDS or AML and died after transplantation or infection (Table 1, P1, P2, P5). In our report, Case 1 was diagnosed early after transient BM failure and successfully underwent HSCT. This indicates that early diagnosis and treatment is important in AMeD syndrome with heterozygous or compound heterozygous *ADH5* and *ALDH2*.

In this report, both cases presented with acquired chromosome 1-related abnormalities. In childhood MDS, monosomy 7 is the most common cytogenetic abnormality that is usually present in approximately 30 % of cases [[6], [7], [8], [9]], whereas gain of the long arm of chromosome 1 (1q) has been considered a rare abnormality [10]. In contrast, FA, a major inherited BM failure with DNA repair abnormalities due to *FANC* abnormalities, the copy number abnormalities that most commonly involve 1q and various partner chromosomes [11] along with complete or partial gain of 1q were found in 52.6 % of FA patients with clonal evolution. In the early stages, *MDM4*, located at 1q, trisomy downregulates p53 signaling and promotes clonogenesis in FA hematopoietic stem or progenitor cells [11]. In AMeD syndrome, elevated formaldehyde has been reported [2] to cause DNA damage and mutational signatures leading to increased risk of cancer. About half of the reported cases of AMeD syndrome also acquired 1q [1,2] (Table 1), which, similar to FA, is presumed to result from early clonal evolution. Therefore, when undiagnosed cases of IBMFS with short stature and excluded FA are found to have acquired 1q, it is necessary to also consider AMeD syndrome.

In summary, the two cases of AMeD syndrome included in this study were successfully treated with HSCT. Both cases acquired 1q as isochromosome 1q. The gain of 1q could be considered as a form of early clonal evolution as in FA and could be useful in diagnosing AMeD syndrome.

## Informed consent

The study was conducted in accordance with the Declaration of Helsinki and approved by the Ethics Committee of the Ehime University. Informed consent was obtained.

## CRediT authorship contribution statement

**Mari Kagajo:** Formal analysis, Validation, Writing – original draft, Writing – review & editing. **Kyoko Moritani:** Conceptualization, Data curation, Writing – review & editing. **Mayumi Iwamoto:** Writing – original draft. **Machiko Miyamoto:** Writing – original draft. **Tsuyoshi Imai:** Conceptualization, Data curation, Writing – original draft. **Motoharu Hamada:** Conceptualization, Data curation, Writing – original draft. **Manabu Wakamatsu:** Data curation. **Hideki Muramatsu:** Conceptualization, Data curation. **Minenori Eguchi-Ishimae:** Conceptualization, Data curation, Formal analysis, Writing – original draft, Writing – review & editing. **Mariko Eguchi:** Conceptualization, Data curation, Writing – original draft, Writing – review & editing.

## Declaration of competing interest

The authors declare that they have no known competing financial interests or personal relationships that could have appeared to influence the work reported in this paper.
